# A rapid enzymatic assay for high-throughput screening of adenosine-producing strains

**DOI:** 10.1111/1751-7915.12189

**Published:** 2015-01-08

**Authors:** Huina Dong, Xin Zu, Ping Zheng, Dawei Zhang

**Affiliations:** 1 Tianjin Institute of Industrial Biotechnology, Chinese Academy of SciencesTianjin, 300308, China; 2 Key Laboratory of Systems Microbial Biotechnology, Chinese Academy of SciencesTianjin, 300308, China; 3 The Light Industry Technology and Engineering, School of Biological Engineering, Dalian Polytechnic UniversityDalian, Liaoning, 116034, China

## Abstract

Adenosine is a major local regulator of tissue function and industrially useful as precursor for the production of medicinal nucleoside substances. High-throughput screening of adenosine overproducers is important for industrial microorganism breeding. An enzymatic assay of adenosine was developed by combined adenosine deaminase (ADA) with indophenol method. The ADA catalyzes the cleavage of adenosine to inosine and NH_3_, the latter can be accurately determined by indophenol method. The assay system was optimized to deliver a good performance and could tolerate the addition of inorganic salts and many nutrition components to the assay mixtures. Adenosine could be accurately determined by this assay using 96-well microplates. Spike and recovery tests showed that this assay can accurately and reproducibly determine increases in adenosine in fermentation broth without any pretreatment to remove proteins and potentially interfering low-molecular-weight molecules. This assay was also applied to high-throughput screening for high adenosine-producing strains. The high selectivity and accuracy of the ADA assay provides rapid and high-throughput analysis of adenosine in large numbers of samples.

## Introduction

Adenosine, an endogenous purine nucleoside, is a conventional drug in the emergency treatment of arrhythmia and drug load test. It antagonizes many of the biochemical and physiological mechanisms implicated in ischemia-reperfusion injury and has been shown to reduce postischemic ventricular dysfunction and myocyte necrosis and apoptosis (Olafsson *et al*., 1987; Kaminski and Proctor, 1989; Meldrum, 1998). It also has been proved to enhance myocardial ischemia tolerance, reduce myocardial reperfusion injury and decrease the infarction area (Lawson *et al*., 1993; Marzilli *et al*., 2000). Meanwhile, adenosine is an important pharmaceutical intermediate that can be used for synthesis of variety of medicinal nucleoside substances, such as adenosine triphosphate (ATP) (Asada *et al*., 1981).

Microbial production of adenosine has drawn more attention recently because of its cost effectiveness and environmentally friendly production process in comparison with chemical production processes. The mass production of adenosine has been focused on the field of microbial production processes development through metabolic engineering and strain breeding. *Bacillus subtilis* is one of the candidates for industrial production of adenosine (Nishiyama *et al*., 1995; Yu *et al*., 2011; Chen *et al*., 2013), which also has a long history as a safe and stable producer of inosine, guanosine and valuable enzymes in commercial processes (Sauer *et al*., 1998; Dong and Zhang, 2014; Zhang *et al*., 2014).

Traditionally, industrial microorganism breeding has been developed via multiple rounds of random mutagenesis by ultraviolet radiation, diethyl sulfate treatment, or low energy ions mutations. The concentration of adenosine was usually measured using High-performance liquid chromatography (HPLC) (Chen *et al*., 2013). HPLC can accurately quantify trace adenosine but require pretreatments to remove proteins or other molecules prior to analysis. Expensive and bulky instruments are required and the samples should be measured one after another. In clinical area, several methods has been developed to determine adenosine in urine or tissues, such as method using reduced S-adenosylhomocysteine hydrolase (Kloor *et al*., 2000), firefly luciferase-based assay (Burgos *et al*., 2012), enzyme- coupled assays (Helenius *et al*., 2012) and aptamer Sensor based methods (Hu *et al*., 2012; Li *et al*., 2012; Wang *et al*., 2012; Fu *et al*., 2013; Zhang *et al*., 2013). However, these methods are not suitable to the detection of large numbers of fermentation samples because of their narrow detection range or high test cost. Therefore, there is an urgent need for the development of accurate and rapid screening method after cell mutagenesis.

An enzymatic assay is one of the promising solutions (Hisamatsu *et al*., 2012) because it can analyze multiple samples simultaneously without any specialized, bulky, and expensive instruments. Adenosine deaminase (ADA; EC 3.5.4.4) participates in purine metabolism where it degrades either adenosine or 2′-deoxyadenosine to inosine or 2′-deoxyinosine, respectively (Eq. 1 and 2).

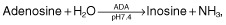
1

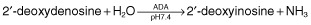
2

To develop a simple and rapid adenosine assay, ADA represented one of the promising enzymes. Several methods have been used to detect the resulting ammonia, such as ion-exchange method (Dienst, 1961; Thomas *et al*., 2002), dry-film method using diffuse separation (Iosefsohn and Hicks, 1985; Diaz *et al*., 1995), indophenol method (Berthelot method) (Ngo *et al*., 1982), microfluorescence assay using phthalaldehyde and mercaptoethanol (Taylor *et al*., 1974; Mroz *et al*., 1982). Enzymatic methods using glutamate dehydrogenase (GLDH) (Talke and Schubert, 1961; Tanganelli *et al*., 1982; da Fonseca-Wollheim and Heinze, 1992), l-glutamine synthetase (GS) (Wakisaka *et al*., 1987) and a enzymatic cycling system composed of three enzymes [NAD synthetase (NADS), glucose dehydrogenase (GlcDH), and diaphorase (DI)] (Yamaguchi *et al*., 2005) have also been developed. In particular, the indophenol method has been widely utilized for clinical and food analyses.

Here we describe an assay method based on ADA to detect adenosine and to improve the efficiency for screening of high adenosine-producing strains. This method combines ADA with indophenol method. The variation of resulting blue color can be monitored via OD_697_ and high-throughput screening can be achieved using 96-well plates. The ADA assay was successfully applied to measure adenosine in broth of adenosine-producing *B. subtilis* strain and verified by HPLC evaluation. The high-throughput screening of adenosine-producing strain was also discussed.

## Results

### Expression and purification of ADA in *Escherichia coli*

**Figure 1:**
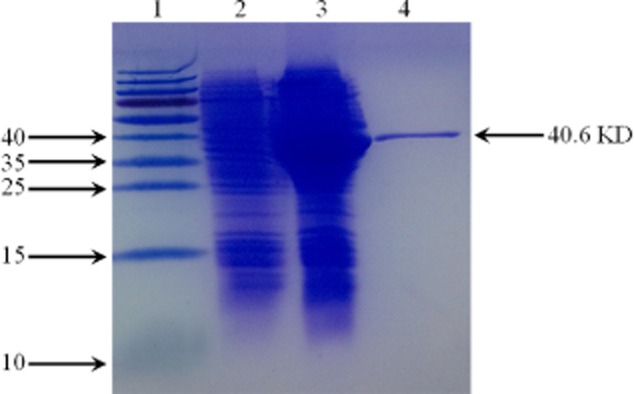
SDS-PAGE analysis of the ADA expression. *E. coli* BL21(DE3) cells containing pET-28a-*add* were grown and induced with 1 mM IPTG. The cells were sonicated and then centrifuged to divide into two fractions, soluble and insoluble fractions. Soluble fractions were then purified using Ni-NTA agarose. Lane 1, size markers; Lane 2, total proteins of the uninduced cells; Lane 3, total proteins of the IPTG-induced cells; Lane 4, purified protein of ADA.

As shown in Fig. 1, adenosine deaminase gene was amplified from *E. coli* 1655 genomic DNA and inserted into pET28a vector, yielding pET28a-*add*. The pET28a-*add* was expressed in *E. coli* BL21(DE3) and induced by IPTG. The induced protein migrated as a 40.6 kDa protein on SDS-PAGE gel. It was shown that most of the induced protein was soluble after purification. The specific activity of purified ADA was estimated to be 15.5 U/mg. Purified ADA was used for the construction of enzymatic assays to detect adenosine as below.

### Activity determination of ADA

The activity determination of ADA was conducted. The expressed ADA has a strong deamination activity to adenosine in comparison with the control group, which lacks ADA. Although blank samples generated a little background signal, the expressed ADA still showed a distinguished activity.

### Determination of adenosine based on ADA

**Figure 2:**
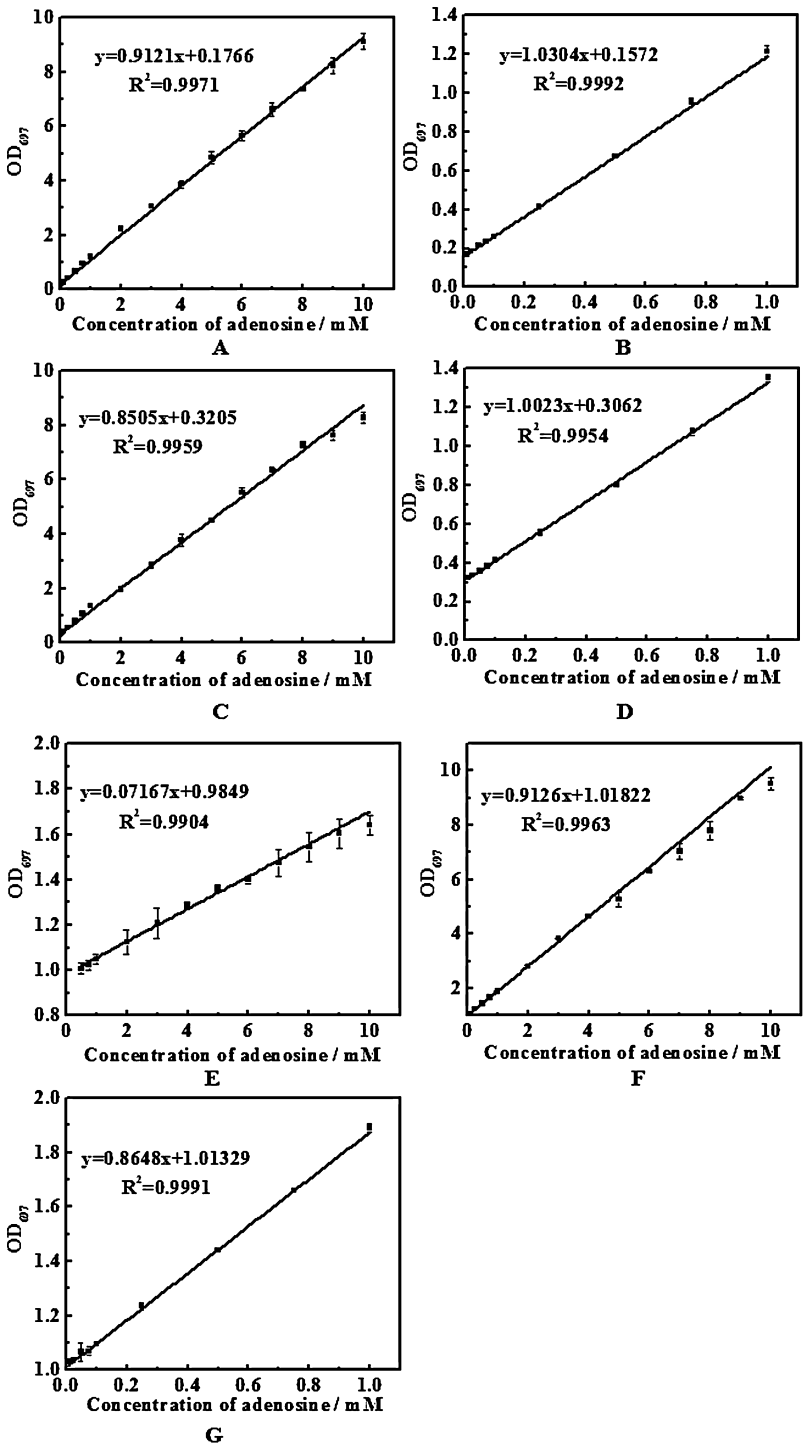
Adenosine standard curves using ADA assay in H_2_O (A and B), modified M9 medium (C and D), LB medium (E) and 10-fold diluted LB medium (F and G), respectively. Each plot represents the average of three samples. Absorbance was measured using a micro-plate reader.

The adenosine assay was developed by coupling ADA to indophenol method. The resulting indophenol has a maximum absorption at 697 nm. The addition of adenosine resulted in a proportional color development giving a linear standard curve (Fig. 2). The linear range and detection limit in H_2_O, LB and M9 media are listed in Table 1. We also showed Signal to background ratios (S/B) generated by the standard curves in different media to present the sensitivity of the ADA method for broth detection in Table 1. The regular M9 medium contains (NH_4_)_2_SO_4_, in which the concentration of NH_4_^+^ was higher than that produced in ADA reaction. The regular M9 has a significant influence on ADA reaction (Fig. S1). Therefore, we replaced it with urea. The modified M9 medium enabled the highly sensitive detection of a low concentration of adenosine. The LB medium has negative influence on the assay, while the influence will significantly decrease as the medium was diluted 10-fold (Fig. 2F and G). Therefore, the fermentation samples in LB medium should be diluted before ADA reaction and the dilution step is a necessary step when LB is used.

**Table 1:** Summary of parameters of the adenosine assay by ADA

	H_2_O	M9 medium	LB medium	10 fold diluted LB medium
Linear range of adenosine concentrations (mM)	0.065–10	0.054–10	3.2–10	0.098–10
Detection limit (mM)	0.065	0.054	3.2	0.098
Z′(calculated with 1 and 10 mM)	0.881	0.911	0.652	0.902
Concentration of adenosine/mM	S/B(H_2_O)	S/B(M9 medium)	S/B(LB medium)	S/B(10-fold diluted LB medium)
0.01	1.08	2.76		1.03
0.025	1.19	2.90		1.03
0.05	1.38	3.08		1.07
0.075	1.51	3.29		1.07
0.1	1.66	3.56		1.08
0.25	2.66	4.80		1.23
0.5	4.31	6.88	1.44	1.44
0.75	6.14	9.27	1.46	1.66
1	7.79	11.65	1.50	1.89
2	14.37	16.97	1.61	2.82
3	19.59	24.69	1.73	3.83
4	24.80	32.62	1.84	4.64
5	31.15	38.84	1.95	5.27
6	36.31	47.54	2.01	6.29
7	42.47	54.51	2.11	7.02
8	47.33	62.92	2.21	7.79
9	52.85	65.68	2.30	8.97
10	58.54	71.32	2.35	9.51

### Substrate specificity test of ADA

The deamination activity of ADA was examined with various nucIeotide-related substances including adenosine, 2′-deoxyadenosine, cytidine, uridine, thymidine, guanosine, adenine, inosine, ATP, ADP, AMP and IMP. The adenosine deaminase catalyzed the deamination of deoxyadenosine besides adenosine And the absorbance of 2′-deoxyadenosine was 1.15-fold of adenosine. It revealed no deamination activity with other kinds of ribonucleosides, especially AMP and IMP, which are the by-products in the fermentation of adenosine-producing strain (Yu *et al*., 2011). Adenine arabinoside, 3′-deoxyadenosine and 2′-deoxyadenosine are the alternative substrates for adenosine (Nygaard, 1978). In general, the enzymatic steps in the de novo biosynthetic pathways of pyrimidine nucleotides are regulated in vivo by feedback inhibition of key enzymes, and by repression and/or attenuation of enzyme synthesis by the accumulation of end products or other metabolites (Roland *et al*., 1985). Accordingly, pyrimidine nucleosides such as deoxyadenosine, which could theoretically be synthesized from the end products (pyrimidine nucleotides), are almost impossible to secrete out of cells and accumulate in media. Thus, ADA could be used to determine specifically the amount of adenosine in fermentation broth.

### Effect of medium components on the adenosine assay

A number of compounds including common media components, some precursors and by-products of adenosine production, were tested for possible interference to ADA assay (Table 2). It is shown that most of components do not interfere the assay. The xylose strongly suppress the development of blue color. The multiple nutrition components including beef power, tryptone, yeast extract and yeast power had some influence on the accuracy of ADA assay (126.5% ∼ 225.4%). The error may be caused by endogenous components with amino functional groups contained in these complicated components. Zn^2+^, Co^2+^, Ca^2+^ and Mn^2+^ cause an increase in absorbance at high concentration, however, when they were at low concentration the influence will decrease (data not shown). The influence of metal ions contained in medium can be ignored as their concentration were lower compared with the experimental concentration.

**Table 2:** Effect of medium components on adenosine assay

compound	Concentration (mM)	% absorbance	compound	Concentration (g/l)	% absorbance
**Mineral salts**			**Nutritional components**		
NaCl	10	98.3	Beef powder	5	225.4
ZnCl_2_	10	124.5	Tryptone	5	137.2
CoCl_2_	10	120.9	Yeast extract	5	126.5
FeCl_3_	10	108.2	Yeast powder	5	219.7
CuCl_2_	10	90.9	Lactose	10	92.0
CaSO_4_	10	117.9	Maltose	10	104.1
MnSO_4_	10	125.8	Sucrose	10	95.7
Na_2_SO_4_	10	94.3	D-glucose	10	88.5
FeSO_4_	10	102.0	D-xylose	10	60.9
CuSO_4_	10	87.6	L-arabinose	10	98.2
Sodium acetate	10	96.5	D-Mannitol	10	99.9
NaHCO_3_	10	72.1	D-Sorbitol	10	95.2
NaNO_2_	10	73.2	Urea	10	106.0
Sodium citrate	10	91.3	Betaine	10	96.4
Calcium carbonate	10	94.4	Tryptophan	2	110.6
sodium pyruvate	10	94.1	AMP	2	108.5
sodium lactate	10	97.4			
M9		102.9	LB		67.5
			10-fold diluted LB		101.9

Note: Values reported in the table were the average of three parallel determinations. The absorbance was reported as a percentage of that obtained with adenosine, (2 mM) dissolved in water, i.e., [(absorbance with adenosine + test compound)/absorbance with adenosine alone] × 100%. A value of 100 means no interference; a value of 0 means total interference, i.e., no color formation at all, and values greater than 100 mean the test compound enhances the absorbance of the solution.

In the above experiments, each component is separately tested in water, the M9 and LB media are the examples (Table 2) to show the extent of interference from real media situation. Additive and synergetic effects could potentially happen when several of these molecules are together. The result showed that the LB medium had inhibition on the ADA assay. The diluted LB in Fig. 2 had decreased inhibition on ADA assay, thus the fermentation broth should be diluted before ADA assay when complex media are used.

### Spike and recovery test with fermentation broth

**Figure 3:**
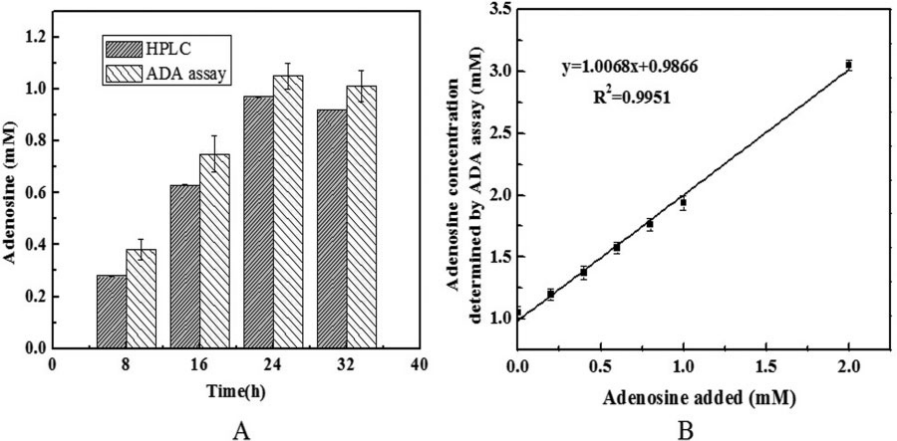
A. Adenosine concentration in the fermentation flask determined using the CDA assay and HPLC.B. Correlation between the enzymatic determination of adenosine and adenosine added concentration in fermentation broth.Each experiment run in triplicate.

To verify whether the ADA assay was applicable to detect the increase of adenosine in fermentation broth, a time course of adenosine production by adenosine-producing strain was shown in Fig. 3A. The results showed that the ADA assay fit well with HPLC method. Considering fermentation time, and the by-products and other metabolites produced in fermentation, fermentation samples at 24 h were chosen to detect adenosine for screening adenosine-production strains.

The reliability of the ADA assay was further supported by the assay results of adding 0–2 mM adenosine to the fermentation broth at 24 h. The assay estimated the intrinsic adenosine concentration in the fermentation sample to be 1.05 ± 0.05 mM. This value was consistent with that estimated by instrumental analysis (HPLC), 0.97 ± 0.002 mM, confirming the accuracy of the ADA assay in biological samples. As shown in Fig. 3B, good linearity (R^2^ = 0.9951) was obtained between the concentration of adenosine added in fermentation broth. From the slope of linear correlation, the recovery of adenosine by ADA determination was 100.7%. These results indicate that an increase in adenosine can be accurately and reproducibly detected by the ADA assay.

### Screening of adenosine-producing strains

To verify whether this ADA assay can be used to quantify the amount of adenosine produced by different bacterial strains at once, a adenosine-producing strain was treated with 402 nm laser for 3 min, isolated on LB agar plate, and then 95 randomly picked colonies were cultured in 96-well deep-hole culture plate with control strains. These strains were then screened using ADA assay with 96-well plate.

**Figure 4:**
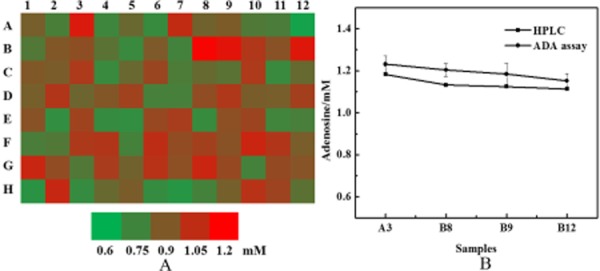
Screening high adenosine-producing strains using the ADA assay.A. Production of adenosine in a 96-well culture plate from randomly picked mutation strains. A1 represented for adenosine concentration of parent strain.B. Top four samples (B8, B9, B12 and A3) determined using the ADA assay were chosen to detect adenosine by HPLC. Each experiment run in triplicate.

The results were presented in Fig. 4 as a heat map and the data were shown in Table S2. The top four adenosine-producing mutants (B8, B9, B12 and A3) chosen by ADA assay were further tested by HPLC (Fig. 4B). The values of ADA assay and HPLC method has positively correlation for adenosine concentration. Though the values from ADA assay in 96-well plate deviated slightly from those determined by HPLC, the ADA assay is still a good method to exclude more than 95% of low-yielding strains.

The suitability of ADA assay for high-throughput screening (HTS) was also estimated. The screening window coefficient (Z′ factor) for this assay was 0.911 and 0.902 in M9 and diluted LB media (Table 1). These high values (maximum of 1.00 for a perfect assay) reflect the overall quality of this assay.

## Discussion

An easy and simple assay for adenosine in this study was developed by coupling ADA and indophenol method to screen and isolate high adenosine-producing bacteria. The enzyme (ADA) constituting the assay can be readily obtained by over-expression in *E. coli*. The assay is a simple detection method as it only requires ADA in the assay mixture. It also has low detection limit, and is highly sensitive to low concentrations of substrate. This assay can be applied to analysis biological samples and allows easier and simpler measurements of adenosine in fermentation broth without any pretreatment. In comparison with the traditional qualitative and quantitative detection of adenosine using chromatography methods such as HPLC, which are expensive, laborious and low-throughput (10^2^–10^4^ colonies per week), the reported assay is high-throughput, quick, sensitive and highly adaptable systems (10^6^–10^8^ colonies per week) (Strege, 1999; Pham-Tuan *et al*., 2003). The methods developed for determination of adenosine in serum, urine or tissue (Kloor *et al*., 2000; Burgos *et al*., 2012; Helenius *et al*., 2012; Li *et al*., 2012; Wang *et al*., 2012; Zhang *et al*., 2013) have nice detection limit and linear relationship. However, the detection range is too narrow (see Table S1) and the test cost is too high for them to be used in detecting the adenosine in fermentation broth in large numbers of samples.

The cultivation medium and the metabolism by-products of the adenosine- producing strains are main factors that might affect the ADA assay to analyze and screen for a higher adenosine-producing strain. The result showed that modified M9 salt-based minimal medium has little influence on the ADA assay, while LB medium has a significant effect on the assay (Fig. 2). The adenosine in M9 medium should be detectable in the range of 0.054–10 mM. The sensitivity of this assay will decrease when adenosine is detected in complex medium containing yeast extract, peptone, tryptone or beef powder. As they all contained compounds with primary or secondary amino groups which strongly suppress the development of blue color in indophenol method (Ngo *et al*., 1982). However, the diluted complex medium had little effect on adenosine assay. Four main by-products, sodium pyruvate, sodium lactate, AMP and IMP, were investigated and it turned out that all of them had no effect on ADA assay. The adenosine also had no influence on ADA assay. However, some other metabolic substances have effect on the assay, for example, the ammonia contained in the fermentation broth samples. Therefore, the blank control experiments should be done to eliminate the background interference. As the by-products accumulated in fermentation will interfere the assay, an appropriate fermentation time (24 h) should be choose to screen adenosine-producing strains. This can eliminate the influence and at the mean time shorten the screening period.

Although the ADA was coupled to indophenol method, the coupling partner could be many other enzymes (e.g. GLDH) or methods that can determine ammonia concentration. These assays can also be used to quantify other biomolecules based on deaminase. Deoxyadenosine and adenosine are equally good substrates for ADA, which means the methods based on ADA are also suitable for high-throughput screening of deoxyadenosine-producing strains.

## Experimental procedures

### Materials and equipments

All chemical reagents were of analytical grade and purchased from Sigma-Aldrich (St Louis, MO, USA). Primerstar *Taq* polymerase was purchased from Takara and restriction endonuclease, T4 ligase and their corresponding buffers were purchased from New England Biolabs (NEB). Ninety-six-well microplates were purchased from Nunc. Ni-NTA agarose resins were supplied by GE Healthcare for His-tagged protein purification.

All polymerase chain reactions (PCR) were performed using a thermal cycler (DNA Engine; Bio-Rad, Hercules, CA, USA). Colorimetric assay were measured by a microplate reader (SpectraMax M2e, Molecular Devices, Sunnyvale, CA, USA). HPLC analysis was performed by Agilent 1260 (Agilent Technologies, Waldbronn, Germany).

### Plasmids, bacterial strains and media

Plasmid pET28a was purchased from invitrogen. The host bacterial DH5α and BL21(DE3) were purchased from TransGen Biotech company for the construction, propagation and expression of plasmids. The adenosine-producing *B. subtilis* was reserved in our laboratory.

Luria Broth (LB) medium contains (per liter) 10 g tryptone, 5 g yeast extract, and 10 g NaCl. LB agar plates were prepared by adding 1.5% agar. M9 minimal salts medium contains (per liter) 12.8 g Na_2_HPO_4_·7H_2_O, 3 g KH_2_PO_4_, 0.5 g NaCl, 1 g (NH_2_)_2_CO, 1 mM MgSO_4_·7H_2_O, 0.1 mM CaCl_2_, 0.05 g tryptophan, micronutrient components (1 μM FeSO_4_·7H_2_O, 0.01 μM ZnSO_4_·7H_2_O, 0.08 μM MnCl_2_·4H_2_O, 0.4 μM H_3_BO_4_, 0.03 μM CoCl_2_·6H_2_O, 0.01 μM CuCl_2_·2H_2_O, and 3 nM Na_2_MoO_4_), and appropriate amounts of glucose and antibiotics.

### Plasmid construction

Two primers were employed to amplify *add* gene from genomic DNA of *E. coli* 1655 and designed as follows: Ec-add-F (5′-CGC*GGATCC*ATGATTGATACCACCCTGCC -3′) and Ec-add-R (5′-CCG*GAATTC*TTACTTCGC GGCGACTTTTT-3′). The PCR product was purified from agarose gel, digested with *Bam*HI and *Eco*RI and subsequently ligated into a pET28a vector. *E. coli* DH5α cells with plasmids were cultured aerobically at 37°C in LB medium or on LB agar plates with 50 mg/l Kanamycin. The constructed pET28a-*add* was expressed in *E. coli* BL21(DE3).

### Expression and purification of ADA

For the expression of ADA, *E. coli* BL21 (DE3) including pET28a-*add* plasmid was grown to an OD 600 of 0.6∼0.8 in LB (contained 50 mg/l Kanamycin) and then induced for 2 h by adding 1 mM isopropyl-1-thio-b-D-galactopyranoside (IPTG). Cells expressing ADA were harvested and cell pellet was suspended in the lysis buffer (50 mM NaH_2_PO_4_, pH 8.0, 300 mM NaCl, 10 mM imidazole, 10 mM β-mercaptoethanol), and then disrupted by sonication. The supernatant fraction was subjected to Ni-NTA agarose and equilibrated for 2 h in 4°C. By applying the mixture to the column, the unbound proteins were washed off the column while the bound proteins were eluted by the elute buffer (50 mM NaH_2_PO_4_, pH 8.0, 300 mM NaCl, 250 mM imidazole, 10 mM β-mercaptoethanol). The purified protein were dialyzed against a storage buffer (50 mM NaH_2_PO_4_, pH 8.0 and 1 mM DTT) for overnight in 4°C (Liu *et al*., 2014). The purified enzyme was subsequently mixed with 30% glycerinum and stored in −20°C before utilization.

### ADA assay for adenosine detection

**Figure 5:**
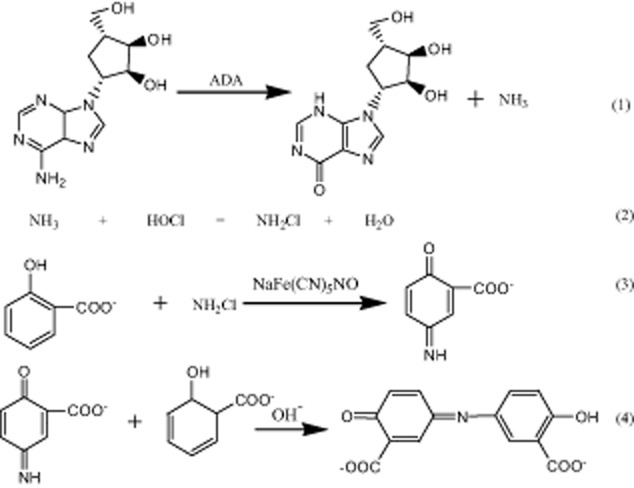
Scheme of the enzymatic assay for adenosine detection at 697 nm.

The featured product of adenosine assay used in this study is indophenol. The ammonia from the cleavage of adenosine will react with salicylate, hypochlorite and nitroprusside to form a diazonium salt (González-Rodríguez *et al*., 2002) with maximum absorption at 697 nm. The reaction mechanism was shown in Fig. 5.

Standard adenosine was prepared in Milli-Q deonized water as stock solution and the determination was performed in a 96-well plate. The reaction mixture with the total volume of 225 μl. Firstly, 25 μl of adenosine samples were mixed with 100 μl 0.01 M phosphate-buffered saline (PBS) and appropriate amounts of the enzyme. The mixture was incubated for 20 min at 37°C, 50 μl reagent I (containing 68 g/l salicylic acid, 25 g/l sodium hydroxide and 2.2 g/l sodium nitroprusside) and 50 μl reagent II (containing 40.9 ml/l sodium hypochlorite ) were added to the above mixture and incubated for 30 min at 37°C. Then, the reaction mixture was diluted in proper ratio with water and measured at 697 nm using a microplate reader. The amount of salicylic acid, sodium hydroxide, sodium nitroprusside and sodium hypochlorite were optimized to improve the sensitivity of the assay. Absorbance measured at the end of the reaction was used to construct adenosine standard curves. The detection limit of the assay was defined as 3 times the standard deviation of adenosine-free blank samples (*n* = 20) (Kameya *et al*., 2014).

### Substrate specificity for ADA assay

The effects of different ribonucleosides on ADA assay were examined. The reaction contained 2 mM ribonucleosides and performed as described above.

### Fermentation and analysis of adenosine production

To determine whether this assay could be applied to the fermentation industry as a rapid and accurate tool for adenosine measuring, salt irons, medium nutrients which are commonly used in conventional microbiological culture media were examined to investigate their effects on the ADA assay. The reaction contained 2 mM adenosine and the assay was performed as described above.

The fed-batch fermentation was also processed in M9 minimal salts medium adding 4% glucose. The fermentation was performed in 250 ml Erlenmeyer flask containing 50 ml M9 medium. The flask was kept in a shaker incubator at 220 rpm and 37°C for 48 h. The biomass concentration was determined by the OD at 600 nm in a UV spectrophotometer. The concentration of adenosine in the cell free culture supernatant was measured using the above assay and HPLC. Samples, harvested at certain time of fermentation, were centrifuged at 13 000 rpm for 2 min at 20°C. The supernatants were filtered through a 0.22 μm membrane filter. An aliquot (10 μl) was injected and analyzed by the Agilent 1260 HPLC with a 5C18-250A column (Agilent, 4.6 mm id × 250 mm) thermostated at 35°C to separate the compounds. The mobile phase consists of water: acetonitrile (96:5 v/v) at a flow rate of 0.8 ml/min and the analytes were detected at 280 nm.

### Z′ Factor

The screening window coefficient was determined as previously described (Zhang, 1999) using two extremes of the standard curve in different media. The following equation was used to calculate the corresponding factor Z′:

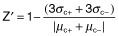
3where μ_c+_ and μ_c−_ are the mean value of absorbance of two extremes of the standard curve and σ_c+_ and σ_c−_ are the standard deviation of the absorbance (with 99.73% confidence limit), respectively.

## Conflict of interest

This work has been included in a patent application by Tianjin Institute of Industrial Biotechnology, Chinese Academy of Science.

## Supporting Information

**Fig. S1.** The determination of different concentrations of adenosine using ADA assay in regular M9 medium. **Table S1.** Comparison of detection range of previous published methods. **Table S2.** Adenosine production of screening strains in 96-well plate.
