# Supplementary material for: A rapid enzymatic assay for high-throughput screening of adenosine-producing strains
Source: Microb Biotechnol. 2015 Jan 8;8(2):230–8. doi: 10.1111/1751-7915.12189 (PMC4353337; doi:10.1111/1751-7915.12189)
Supplement: Supplementary file 1 [file mbt20008-0230-sd1.doc]

**Fig. S1** The determination of different concentrations of adenosine using ADA assay in regular M9 medium

**Table S1 Comparison of detection range of previous published methods**

| Methods | detection range | reference |
| --- | --- | --- |
| Adenosine-binding protein assay | 1-1000pM |  |
| Luciferase-Based Assay | 1−80 pM |  |
| Enzyme-coupled fluorometric purine- sensing assay | 5-400 nM |  |
| Luminescent aptamer biosensor- Based Assay | 0.06-1mM |  |
| Label-free bifunctional electrochemiluminescence aptasensor-Based Assay | 0.5-7nM |  |
| Time-resolved fluorescence sensor-Based Assay | 0.5-100 nM |  |

**Table S2** Adenosine production of screening strains in 96-well plate

| mM | 1 | 2 | 3 | 4 | 5 | 6 | 7 | 8 | 9 | 10 | 11 | 12 |
| --- | --- | --- | --- | --- | --- | --- | --- | --- | --- | --- | --- | --- |
| A | 0.878 | 0.773 | 1.114 | 0.747 | 0.846 | 0.729 | 1.056 | 0.868 | 0.893 | 0.789 | 0.779 | 0.649 |
| B | 0.793 | 0.905 | 0.936 | 0.812 | 0.736 | 0.898 | 0.757 | 1.183 | 1.132 | 1.015 | 0.922 | 1.124 |
| C | 0.903 | 0.887 | 1.005 | 0.736 | 0.847 | 0.967 | 0.782 | 0.841 | 0.812 | 1.005 | 0.736 | 0.84 |
| D | 0.878 | 1.018 | 0.857 | 0.908 | 0.978 | 0.767 | 0.766 | 0.941 | 1.01 | 0.883 | 0.88 | 0.986 |
| E | 0.922 | 0.719 | 0.975 | 0.727 | 0.713 | 0.956 | 0.995 | 0.729 | 0.93 | 0.969 | 0.753 | 0.77 |
| F | 0.775 | 0.791 | 1.007 | 1.017 | 0.761 | 1.047 | 0.944 | 0.982 | 0.921 | 1.074 | 1.017 | 0.886 |
| G | 1.073 | 0.942 | 0.792 | 0.997 | 0.866 | 1.031 | 0.934 | 1.076 | 0.953 | 0.762 | 0.948 | 0.917 |
| H | 0.699 | 1.068 | 0.725 | 0.843 | 0.887 | 0.726 | 0.692 | 0.761 | 0.876 | 1.032 | 0.974 | 0.816 |

**Reference**

1 Kloor, D., Yao, K., Delabar, U., and Osswald, H. (2000) Simple and Sensitive Binding Assay for Measurement of Adenosine Using Reduced S-Adenosylhomocysteine Hydrolase, *Clin Chem* **16**: 537–542.

2 Burgos, E.S., Gulab, S.A., Cassera, M.B., and Schramm, V.L. (2012) Luciferase-Based Assay for Adenosine: Application to S-Adenosyl-l-homocysteine Hydrolase, *Anal Chem* **84**: 3593-3598.

3 Helenius, M., Jalkanen, S., and Yegutkin, G.G. (2012) Enzyme-coupled assays for simultaneous detection of nanomolar ATP, ADP, AMP, adenosine, inosine and pyrophosphate concentrations in extracellular fluids, *Biochim Biophys Acta.* **1823**: 1967-1975.

4 Li, L.-L., Ge, P., Selvin, P.R., and Lu, Y. (2012) Direct Detection of Adenosine in Undiluted Serum Using a Luminescent Aptamer Sensor Attached to a Terbium Complex, *Anal Chem* **84**: 7852-7856.

5 Wang, H., Gong, W., Tan, Z., Yin, X., and Wang, L. (2012) Label-free bifunctional electrochemiluminescence aptasensor for detection of adenosine and lysozyme, *Electrochim Acta* **76**: 416-423.

6 Zhang, K., Wang, K., Xie, M., Xu, L., Zhu, X., Pan, S., et al. (2013) A new method for the detection of adenosine based on time-resolved fluorescence sensor, *Biosens Bioelectron* **49**: 226-230.
